# Supplementary material for: Assessment of Innovative Dry Powders for Inhalation of a Synergistic Combination Against Mycobacterium tuberculosis in Infected Macrophages and Mice
Source: Pharmaceutics. 2025 May 27;17(6):705. doi: 10.3390/pharmaceutics17060705 (PMC12195745; doi:10.3390/pharmaceutics17060705)
Supplement: Supplementary file 1 [file pharmaceutics-17-00705-s001.zip › pharmaceutics-3617974-supplementary.pdf]

Supplementary data

S1. Materials.

VAN and THL were purchased from Cayman Chemicals (Ann Arbor, USA). HCO Kolliwax and Poloxamer 407 were purchased from BASF (Ludwigshafen, Germany). Monocytes THP-1 (TIB-202) cells were purchased from ATCC (Manassas, USA). Roswell Park Memorial Institute (RPMI) 1640 medium was purchased from Carl Roth (Karlsruhe, Germany). Phosphate buffer saline (PBS), foetal bovine serum (FBS), albumin-dextrose complex (ADC), and the oleic-albumin-dextrose (OADC) complex were purchased from Gibco and Difco Laboratories (Thermofischer, Dilbeek, Belgium). Ethanol absolute and sterile syringe with cellulose filter (0.2 µm pore diameter) were purchased from VWR (Radnor, USA). Phorbol 12-myristate 13 acetate (PMA) were purchased from Promega (Madison, USA). Rifampicin, amikacin, Tween 80, carboxymethylcellulose (CMC) and methyl acetate were purchased from Sigma Aldrich (Saint-Louis, USA). ZAP-OGLOBIN II Lytic Reagent was purchased from Beckman Coulter (Nyon, Switzerland). MB/BacT antibiotic supplement was purchased from BioMérieux (Craponne, France). The monohydrate lactose LH300 (585814) was purchased from DOMO (Goch, Germany). The food and the Solid Drink® for mice were purchased from Carfil Quality (Oud-Turnhout, Belgium). The 90 mm glass fibber filter (1 µm pore size) was purchased from Pall Corporation (New York, USA). Size 3 hydroxypropyl methylcellulose (HPMC) Quali-V Hypromellose capsule was purchased from Qualicaps (Madrid, Spain). The 0.7 µm pore size polycarbonate filter was purchased from Agilent Technologies (Machelen, Belgium). TZERO hermetic aluminium pan and TZERO hermetic lid were purchased from TA Instruments (New Castle, USA). Ultrapure Mili-Q water was generated from a Pure-Lab Ultra purification system (Elgan, Lane End, UK). All solvents used were analytical grade.

S2. Analytical validation of the High performance liquid chromatography connected to diode array detector (HPLC-DAD) method.

S2.1. Method

Assay validation of the HPLC-DAD method developed was carried out according to the International Conference on Harmonization guidelines [45]. The stock standard solutions were prepared by dissolving approximately 20.0 mg of VAN or THL, accurately weighted, in 4.0 mL of the dilution phase (a mixture of ultrapure water and absolute ethanol (75:25 *v/v*)) to produce a final concentration stock solution of about 5 mg/mL. Standard solutions of VAN (approximately 50, 100, 150, and 200 µg/mL) and THL (approximately 5, 10, 15, and 20 µg/mL) were prepared by appropriate dilution of the stock standard solutions. The standard solutions were sonicated, heated at 35 °C for 30 minutes, and then filtered with a 0.2 µm polycarbonate filter before placing them in vials for HPLC analysis.

To validate the method, three independent series of standard calibration solutions in the range of about 50-200 µg/mL for VAN and about 5-20 µg/mL for THL were prepared and analysed. Calibration curves were constructed by plotting the measured peak area of VAN and THL against the concentrations of the standard solutions. The method was analysed including linear function, coefficient of linear regression and linearity range. To establish the within-day and between-day accuracy and precision of the method, three replicates of standard solutions at four different concentrations were assayed on one day and three separate days.

S2.2. Results and discussion

The development of a fast, accurate and precise method for quantification of both VAN and THL contents in VAN/THL formulations was necessary for drug quality control during production and characterization. Calibration curves were constructed using three series of standard VAN and THL solutions in the range of 50-200 µg/mL and 5-20 µg/mL respectively. Under the described chromatographic conditions, VAN and THL are eluted at about 5 and 14 min, respectively. The equations of linear regression and statistical data are presented in Table S1. The linearity of the calibration curve is validated by a determination coefficient value above 0.999.

Table S1. Statistical data of calibration curves of VAN and THL.

Parameter	VAN	THL
**Concentration range**	50-200 µg/mL	5-20 µg/mL
**Regression equation**	$y = 11.717x – 5.8132$	$y = 126.4x + 2.057$
**Coefficient of determination (r^2)**	0.9997	0.9999
**Standard deviation of slope**	0.2	8.6
**Relative standard deviation of slope (%)**	1.7	6.8

The accuracy and precision were determined by analysing three samples of VAN at approximately 50, 100, 150, and 200 µg/mL and THL at approximately 5, 10, 15, and 20 µg/mL on three separate days. Concentrations were determined using calibration standard curves prepared for each day (Table S1). Good accuracy and repeatability were observed over the entire concentration range. The within-day and between-day variability demonstrated CV values less than 0.71 % at all four selected concentrations (Table S2).

Table S2. Accuracy and precision of method for determination of VAN and THL contents.

VAN			THL		
**Concentration added (µg/mL)**	**Concentration found (mean ± SD) (µg/mL)**	**CV (%)**	**Concentration added (µg/mL)**	**Concentration found (mean ± SD) (µg/mL)**	**CV (%)**
Within-day (n = 3)			Within-day (n = 3)		
**50**	51.2 ± 0.4	0.71	**5**	5.1 ± 0.03	0.51
**100**	102.0 ± 0.2	0.25	**10**	10.1 ± 0.06	0.59
**150**	152.8 ± 0.2	0.11	**15**	15.2 ± 0.09	0.57
**200**	203.6 ± 0.3	0.17	**20**	20.3 ± 0.1	0.53
Between-day (n = 9)			Between-day (n = 9)		
**50**	51.1 ± 0.3	0.61	**5**	5.1 ± 0.03	0.53
**100**	102.2 ± 0.5	0.52	**10**	10.1 ± 0.06	0.56
**150**	153.2 ± 0.8	0.53	**15**	15.2 ± 0.08	0.55
**200**	204 ± 1	0.55	**20**	20.3 ± 0.1	0.52

S2.3. Conclusion

The developed HPLC method provides a simple and accurate quantitative approach for routine *in vitro* tests of VAN and THL dosage forms. It is suitable for determination of VAN and THL content in simple matrix samples.

S3. Physicochemical properties of raw materials, SD VAN and dry powders for inhalation

S3.1. Methods

- *Residual solvent content*

The residual solvent content was evaluated using thermogravimetric analysis (TGA) using a Q500 thermogravimetric analyser (Thermal Analysis (TA) Instruments, New Castle, USA) and Universal Analysis 2000 software version 4.5A (TA Instruments, New Castle, USA). Runs were done in triplicate on 5-7 mg of sample, set with platinum pan from 30 to 200 °C at a heating rate of 10 °C/min. Weight losses were assessed between 35 and 105 °C to determine the total residual solvent content using Universal Analysis 2000 software v. 4.5A.

- *The geometrical particle size distribution (PSD)*

The PSD was measured as suspended and individualized particles in VAN-saturated isopropanol containing 0.1 % w/v Poloxamer 407. This analysis was done using a Mastersizer 3000 laser diffractometer (Malvern Instruments Ltd., Worcestershire, UK) connected to a Hydro MV dispenser equipped with a 40 W ultrasonic probe (Malvern Instruments Ltd., Worcestershire, UK). The agitation rate in the Hydro MV disperser was set to 2500 rpm. The analysis was based on the Mie model, appropriated for small particles, and based on both diffraction and diffusion of the light around the particle. The refractive index and absorption coefficient for dispersed VAN was set to 1.74 and 0.1 respectively. The refractive index of dispersant media was set to 1.38 for isopropanol. The PSD was expressed with different parameters: Dv(10), Dv(50), Dv(90). The span was also calculated to determine the width of the PSD.

- *Scanning electron microscopy (SEM) analysis*

The shape, agglomeration state, and surface morphology were evaluated by SEM, using an analytical field emission scanning electron microscope Hitachi SU-8020 SEM-FEG (Chiyoda, Tokyo, Japan). Samples were spread on support via a conductive double-sided sticker and then scattered on graphite. Acceleration during observation was 1 kV. The geometric mean particle diameter of the spray-dried formulations was determined on 300 particles from the SEM images using ImageJ 2.14 software (Image Processing and Analysis in Java).

- *X-ray powder diffraction (XRPD) analysis*

Diffraction patterns were determined using an X-ray diffractometer D8 Advance Eco with a one-dimensional silicon detector Lynxeye XE-T (Brucker, Massachusetts, USA). The source of radiation was Cu Kα. Runs used a 40 kV voltage, a 25-mA current and a scanning rate of 1° per minute.

The percentage of crystalline phase in the sample was evaluated using the surface area ratio with Diffrac Suite™ software (Brucker, Massachusetts, USA). The following equation was used to determine the percentage of crystallinity:

$$\%\,Crystallinity = \left(\frac{AC}{AT}\right) \times 100$$

Where AC is the area under the diffractogram without integrating the deviation from the baseline and AT is the total area under the diffractogram.

- *Differential scanning calorimetry (DSC) analysis*

A Q2000 differential scanning calorimeter (TA Instruments, New Castle, USA) was used to determine thermal properties. A TZERO™ Technology with a RCS90 refrigerated cooling system (TA Instruments, New Castle, USA) was used. Runs were done in triplicate on 2-4 mg of sample, set with a TZERO hermetic aluminium pan sealed with a TZERO hermetic lid to be submitted to 3 cycles of heat/cool rate set at 10 °C/min over a temperature range of –50 °C to 150 °C. A nitrogen atmosphere was applied to analyse samples, and an empty sealed pan was used as a reference. The thermograms were analysed using Universal Analysis 2000 software v. 4.5A (TA Instruments, New Castle, USA).

S3.2. Results

- *Residual moisture/solvent by TGA analysis*

Residual moisture or solvent content determined by TGA just after production (T0) and after 9 months in a desiccator at room temperature are reported in percentage in Table S3 and some illustrative thermograms are presented in Figure S1.

Table S3. Residual moisture/solvent content in % of the spray-dried formulations were determined by TGA over time. Results are expressed as means ± SD (n = 3).

Formulation	T0* (%)	T9* (%)
SD-VAN	8.13 ± 0.02	8.02 ± 0.01
VAN/THL	7.95 ± 0.02	7.89 ± 0.03
VAN/THL/25% HCO	5.39 ± 0.04	5.67 ± 0.01
VAN/THL/50% HCO	4.71 ± 0.02	68. 0.01

*at the day of production (T0) or after 9 months (T9).

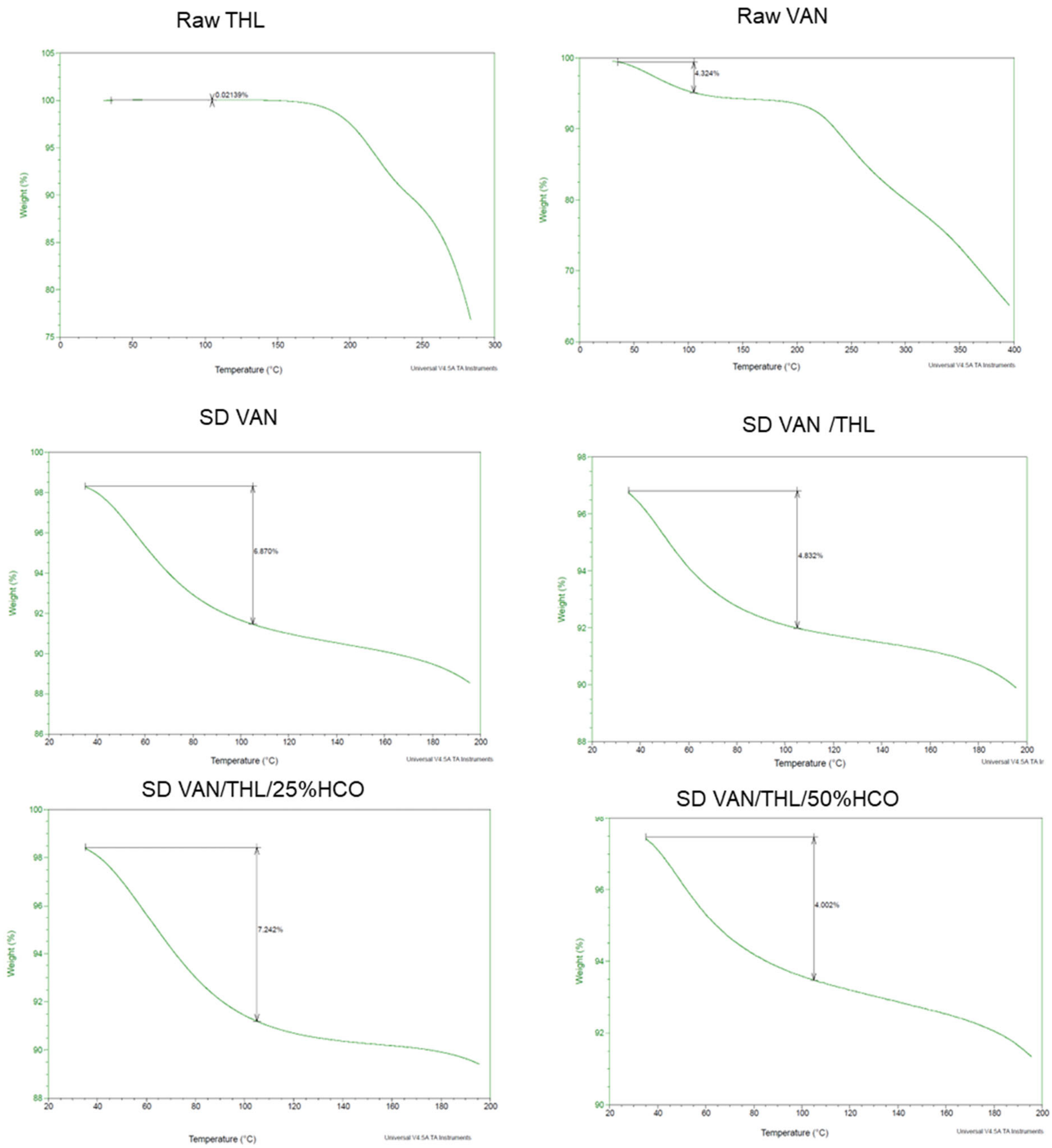

Figure S1. Thermograms of raw VAN, raw THL and the spray-dried formulations SD-VAN, VAN/THL, VAN/THL/25% HCO, VAN/THL/50% HCO. Annotation of the weight loss between 35 and 105°C are made on the figures using the Thermal analysis software.

- *PSD analysis*

Particle size distribution determined by laser diffraction technique just after production (T0) are reported for raw material and spray-dried formulations in Table S4.

Table S4. Particle size distributions of raw VAN, SD-VAN, VAN/THL HCO25%, VAN/THL HCO50% using laser diffraction technique after liquid dispersion. All results are expressed as means in µm ± SD (n = 3).

Samples	Dv10 (µm)	Dv50 (µm)	Dv90 (µm)	Span
Raw VAN	16.1 ± 0.4	76 ± 4	212 ± 16	1.6 ± 0.2
SD-VAN	1.4 ± 0.1	3.0 ± 0.2	5.58 ± 0.09	1.5 ± 0.1
VAN/THL	1.3 ± 0.1	2.7 ± 0.1	5.7± 0.3	1.8 ± 0.1
VAN/THL HCO25%	1.4 ± 0.3	2.8 ± 0.3	5.3± 0.4	1.7 ± 0.3
VAN/THL HCO50%	1.3 ± 0.2	2.8 ± 0.4	4.8± 0.4	1.29 ± 0.06

- *SEM analysis*

The shape, surface morphology, agglomeration state, and size particles of the spray-dried formulations were visualized by SEM (Figure S2).

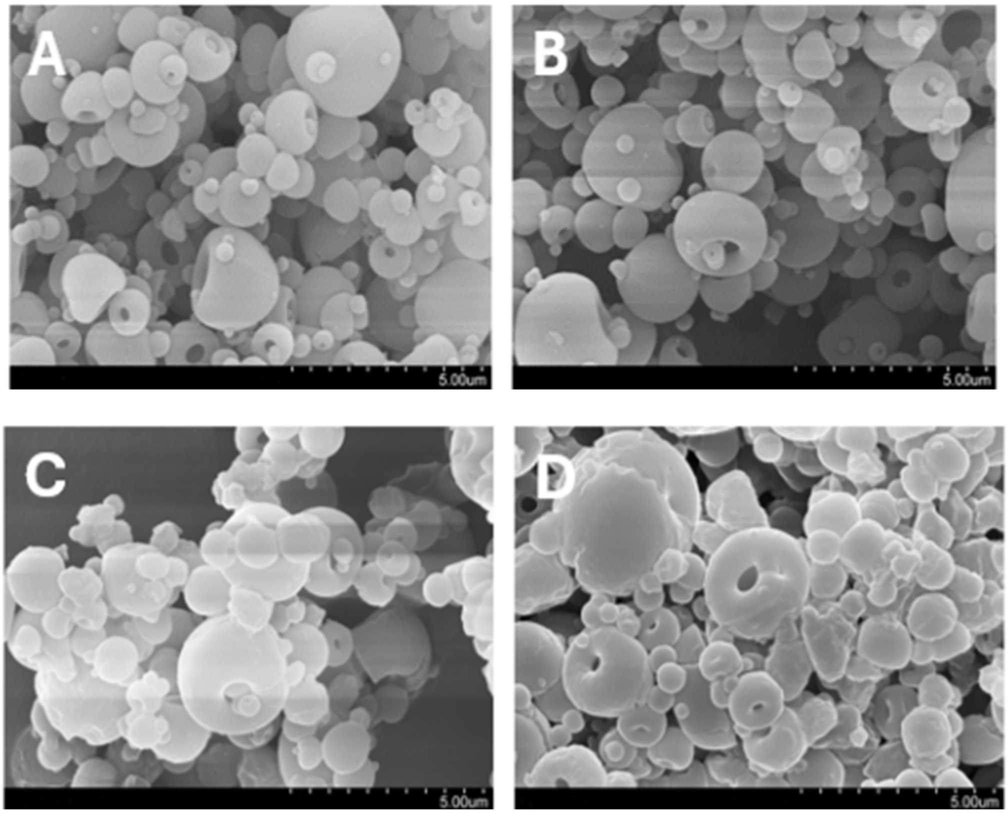

Figure S2. Morphological aspects of the spray-dried formulations. (A) SD-VAN; (B) VAN/THL; (C) VAN/THL/25% HCO; (D) VAN/THL/50% HCO (scanning electron microscopy, magnification 10000x, scale at 5 µm).

The doughnut shape of the dried particles results from the evaporation of the solvent from the droplet and the saturation solubility of the solid material. Diffusion rate of VAN is low compared to water molecule, due to its size. Consequently, water molecules move faster within the droplets than VAN. During the evaporation of water, VAN progressively solidifies but allows some water molecules from the center to pass through the porous shell, leading to the doughnut shape, as reported for nanoparticles [46]. In the second phase of production, the SD VAN in doughnut shape remains in a solid state in the droplets and gave the final form of the dried particles covered with THL and/or lipid HCO.

- *XRPD analysis*

Amorphous content of supplied materials and spray-dried formulations, determined by XRPD analysis just after production (T0) and after 9 months (T9) in a desiccator at room temperature are reported in Table S5.

Table S5. Determination of the amorphous content in % by using X-ray diffraction.

**Component**	**T0 (%)**	**T9 (%)**
Raw VAN	100 ± 0	/
Raw THL	31 ± 0	/
Raw HCO	47 ± 0	/
SD-VAN	100 ± 0	100 ± 0
VAN/THL	97 ± 1	97 ± 2
VAN/THL/25% HCO	93 ± 5	91 ± 2
VAN/THL/50% HCO	89 ± 4	90 ± 1

All results are expressed as means of amorphous content in % ± SD (n = 3).

- *DSC analysis*

Thermal characteristics of supplied materials and spray-dried formulations were determined by DSC. The thermograms are represented in Figure S3 and Figure S4.

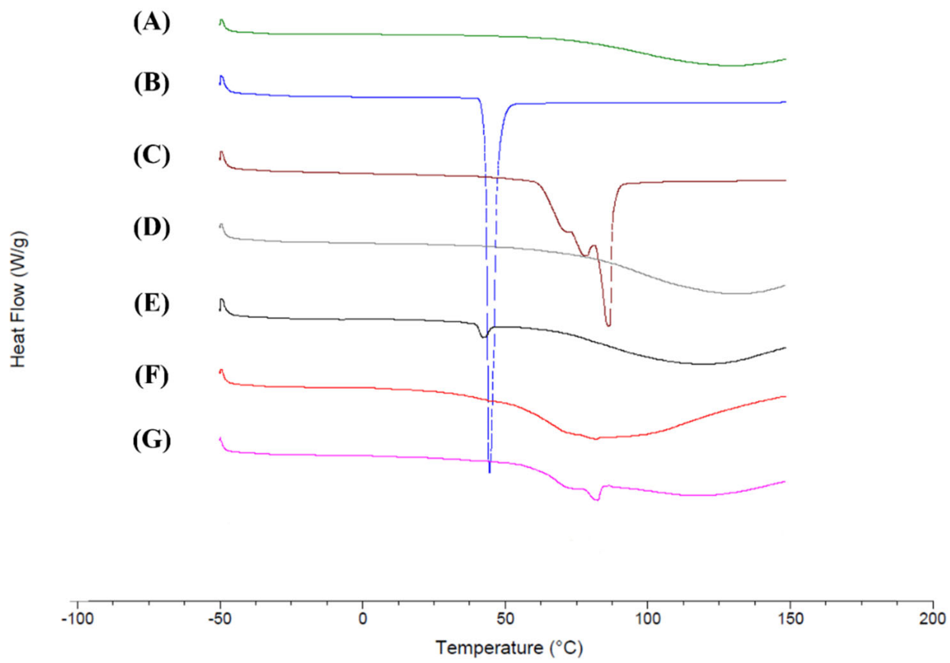

Figure S3. DSC curves of supplied materials. (A) raw VAN, (B) raw THL and (C) raw HCO; and spray-dried formulations: (D) SD-VAN, (E) VAN/THL, (F) VAN/THL/25% HCO, and (G) VAN/THL/50% HCO.

THL melts at 44°C as shown in the DSC curve (Figure S1, S4) and remains stable after reported in DSC curve hereunder of three cycles from 25 to 120°C (cycle 1), 120°C to -50°C (cycle 2) and -50°C to 120°C (cycle 3).

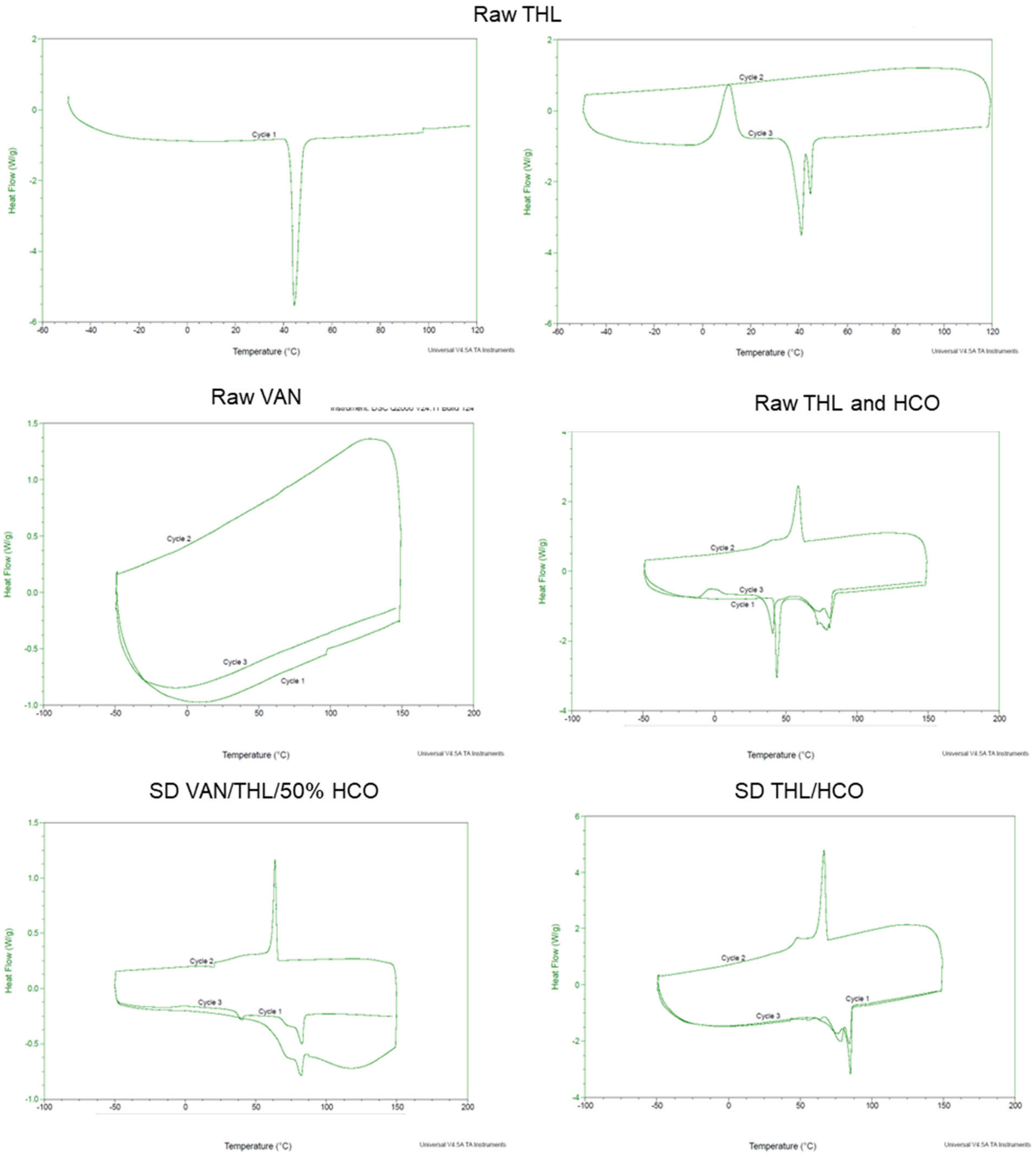

Figure S4. DSC curves of three cycles from -50°C to 120°C (cycle 1), 120°C to -50°C (cycle 2) and -50°C to 120°C (cycle 3) of raw THL, raw VAN, raw THL/HCO, SD THL/HCO, SD VAN THL/50% HCO.

S4. Evaluation of the emission efficiency from blends based on dry powders for inhalation through the PADA insufflator.

S4.1. Methods

- *In vitro emission in mass and dose through the PADA insufflator*

The PADA was used to deliver powder formulations, and the *in vitro* emission efficiency in mass was evaluated on 10 samples. Approximatively 2.00 mg of blend were accurately and precisely weighted to fill the sample chamber of the device. The device was weighed before and after five actuations through the septum of a 10 mL glass vial. The emission efficiency in mass was expressed as the average ratio of emitted powder to the loaded powder from the PADA. The emission efficiency in mass were evaluated by using dilution phase and quantification in the glass vial using the HPLC-DAD method. Uniformity of the delivered mass was expressed as the percentage of the difference from the mean using the coefficient of variation (CV).

To evaluate the emission efficiency in dose of the PADA, 10 samples of blend of an exactly weighed quantity of powder of about 20.00 mg were emitted after five actuations through the septum of a 10 mL glass vial and the emitted dose was then dissolved and dilute in a volumetric flask containing the dilution phase (ultrapure water and absolute ethanol, 75:25 *v/v*). Solutions were sonicated at 35 °C for 30 minutes. The emission efficiency in dose was expressed as the average ratio of the amount of VAN and THL in the delivered mass of powder to the theoretical emitted amount calculated from the emitted mass. The quantification of VAN and THL content in the solution were quantified using the HPLC-DAD method. Uniformity of the delivered dose was expressed as the percentage of the difference from the mean using the CV.

- *Statistical analysis*

The mean of each group ± SD was calculated on at least 3 assays. GraphPad Prism® software (Version 8.4.3) was used to perform statistical analyses. A t-test was applied for comparing 2 groups. An ANOVA and Bonferroni's multiple comparisons tests were applied when comparing more than 2 groups. For dissolution assay, fit factors f_1 and f_2 were calculated. The statistical results were expressed as follow: non-significant for $p > 0.05$, statistically significant (*) for $p < 0.05$, very significant (**) for $p < 0.01$, and extremely significant for $p < 0.001$ (***) and for $p < 0.0001$ (****).

S4.2. Results

- *In vitro emission efficiency in mass and in dose of the blends through PADA*

In vitro emission efficiency in mass of the endotracheal device was statistically better with VAN/THL/25% HCO blends than VAN/THL blends ($p < 0.0001$, ****) (Table S6). For a good uniformity of the delivered mass, the CV should be less than 5 %. VAN/THL/25% HCO and VAN/THL blends demonstrated a higher variability (6.2-6.5%), values that are nonetheless acceptable. *In vitro* emission efficiency of dose of VAN/THL/25% HCO and VAN/THL blends demonstrated that VAN and THL contents were inside the limits of 75 to 125 % demonstrating that uniformity of delivered dose was met for all blends, according to recommendations for human medicine administered by inhalation. Based on these *in vitro* results, VAN/THL and VAN/THL/25% HCO blends demonstrated appropriate characteristic to be administered to mice via the endotracheal powder insufflator.

Table S6. *In vitro* emission efficiency in mass and in dose (in %) from blends based on dry powders for inhalation through the PADA insufflator.

Blend	Emission efficiency in mass (%)	Emission efficiency in dose (%)
**B-VAN/THL/HCO 25%**	94 ± 6 (6)	101 ± 9 (9)
**B-VAN/THL**	77 ± 5 (6)	104 ± 12 (11)

All results are expressed as means ± SD (CV) (n = 10).

S5. Additional information on *in vivo* studies

S5.1. Number of mice used to study VAN/THL efficacy

Two different experiments were performed to study VAN/THL efficacy in vivo. The amount of mice per group included in the experiments are presented in Table S7.

Table S7. Number of mice and treatments used to study VAN/THL *in vivo* efficacy

VAN/THL or VAN/THL/25% HCO based experiments		
**Experiment 1**	**Number of mice/group**	**Treatment duration**
CRT	8	2 weeks
VAN/THL	5	2 weeks
**Experiment 2**	**Number of mice/group**	**Treatment duration**
CRT	14	
HCO	16	2 weeks
VAN/THL/25% HCO	20	2 weeks
**Rifampicin-based experiments**		
**Experiment 1**	**Number of mice/group**	**Treatment duration**
RIF 10 mg/kg	12	1 week
RIF10mg/kg + VAN/THL/25% HCO	12	1 week
**Experiment 2**	**Number of mice/group**	**Treatment duration**
RIF 7.5 mg/kg	10	2 weeks
RIF 7.5 mg/kg+ VAN/THL	10	2 weeks

S5.2. Statistical analysis for in vitro and in vivo efficacy assays

The *p*-values after 24 hours and 4 days treatment during *in vitro* efficacy assay in infected macrophages are reported in Table S7 and Table S8, respectively.

Table S8. *P*-values after 24 hours treatment during *in vitro* efficacy assay in infected macrophages

	**Control**	**HCO**	**VAN/THL/50% HCO**	**VAN/THL/25% HCO**	**VAN/THL**
**Control**	-	p = 0.8043	p < 0.0001 ****	p < 0.0001 ****	p < 0.0001 ****
**HCO**	p = 0.8043	-	p = 0.025 *	p < 0.0001 ****	p < 0.0001 ****
**VAN/THL/50% HCO**	p < 0.0001 ****	p = 0.025 *	-	p = 0.3480	p < 0.0001 ****
**VAN/THL/25% HCO**	p < 0.0001 ****	p < 0.0001 ****	p = 0.3480	-	p = 0.1848

Table S9. *P*-values after 4 days treatment during *in vitro* efficacy assay in infected macrophages

	**Control**	**HCO**	**VAN/THL/50% HCO**	**VAN/THL/25% HCO**	**VAN/THL**
**Control**	-	p = 0.0548	p < 0.0001 ****	p < 0.0001 ****	p < 0.0001 ****
**HCO**	p = 0.0548	-	p < 0.0001 ****	p < 0.0001 ****	p < 0.0001 ****
VAN/THL	p < 0.0001 ****	p < 0.0001 ****	p < 0.0001 ****	p < 0.0001 ****	-
**VAN/THL/50% HCO**	p < 0.0001 ****	p < 0.0001 ****	-	p < 0.0003 ***	p < 0.0001 ****
**VAN/THL/25% HCO**	p < 0.0001 ****	p < 0.0001 ****	p < 0.0003 ***	-	p < 0.0001 ****

The P-values during *in vivo* efficacy assay in Mtb infected mice treated during 2 weeks, three times a week, after one week post infection, are reported in Table S9.

Table S10. *P*-values during *in vivo* efficacy assay in Mtb infected mice with HCO, VAN/THL or VAN/THM/HCO 25% based blends.

	**Control**	**HCO**	**VAN/THL/25% HCO**	**VAN/THL**
**Control**	-	p = 0.1668	p = 0.9002	p = 0.8904
**HCO**	p = 0.1668	-	p = 0.8930	N.D.

N.D. : not determined

S6. In vivo tolerance study with oral rifampicin

S6.1. Methods

Rifampicin (RIF) powder was suspended in a dispersion of 0.5 % CMC and 0.5 % Tween 80 in water. The mixture was vortexed and stirred for 30 min at 50 °C. The suspension was then poured into glass vials at the exact dose (5, 7.5, 10 or 15 mg/kg) and stored at 4 °C refrigerator until use. RIF was administered by gavage using a canula (100 µL per mouse). Preparation of VAN/THL/HCO 25% blend was performed as described in section 2.4.3.

The animal protocol was registered under ethical 727N number (LA1230568) approved on the 12th February 2020. Eight to ten-week-old male (n = 16, approximately 24.6 g) and female (n = 16, approximately 19.5 g) BALB/cAnNRj mice, purchased from Janvier Labs (Le Genest-Saint-Isle, France), were used after a quarantine period of 5 days.

The tolerance study lasted 2 weeks and uses 4 different groups of 6 mice (3 males and 3 females) that were treated by RIF at 5, or 7.5, or 10, or 15 mg/kg by gavage 5 times a week (Monday up to Friday) for two consecutive weeks, and with the blend based on VAN/THL/25% HCO dry powder for inhalation at 500 mg/kg of VAN and 50 mg/kg for THL by endotracheal route using the PADA 3 times a week (Monday, Wednesday, and Friday), after ketamine/xylazine anaesthesia, for two consecutive weeks. Another group of 4 mice (2 males and 2 females) was used as gavage vehicle control and was administered 5 times a week (Monday up to Friday) during two consecutive weeks. Four mice (2 males and 2 females) did not receive any treatment or vehicle and were used as a negative control. Mice were supplemented with Solid Drink® after the first week of treatment.

Mice were euthanized by cervical dislocation after 1-week recovery by Namur Research Institute for Life Sciences. A macroscopic autopsy was performed. Blood was collected by retro-orbital puncture and subjected to biochemical analysis. Blood values for albumin, alkaline phosphatase, alanine aminotransferase, amylase, total bilirubin, urea nitrogen, calcium, phosphorus, creatinine, glucose, sodium, potassium, prothrombin, and globulin were measured. Lungs, livers, and kidney were also collected, washed in fresh PBS solution, and fixed in 10 % buffered formaldehyde solution for 48 h. The organs were then embedded in paraffin wax and stained with haematoxylin and eosinophil (HE) coloration. A periodic acid Schiff (PAS) coloration was also applied for kidneys and livers slides when needed to evaluate any glycogen depletion. The investigation of organ slides (2 tissue sections per organ) was conducted by an independent pathologist, as a randomized and blinded study.

The severity of each observation was scored. For lungs, the score was ranged from 0 to 5 (0: no injury, 1: injury in 1-20 % of the field, 2: injury in 21-40 % of the field, 3: injury in 41-60 % of the field, 4: injury in 61-80 % of the field, 5: injury in > 80 % of the field) for each observation (bronchial epithelial vacuolation (BEV), congestion (Cong), alveolar luminal macrophages (ALM), intra-alveolar fibrin (IAF), intra-alveolar haemorrhage (IAH), pneumocyte hyperplasia (PH), and acute bronchopneumonia (AB)). For livers, the score was ranged from 0 to 3 (0: absent, 1: slight, 2: moderate, 3: severe) for each observation (lobular infiltrate (LI), periportal infiltrate (PI), focal hepatocyte necrosis (FHN), mega-mitochondria (MM), steatosis (Stea), and intranuclear glycogen inclusion (IGI)). Glycogen depletion (GD) was determined by PAS coloration [47]. For kidneys, tubular necrosis (TN) was determined with a severity score ranging from 0 to 2 (0: absent, 1: focal, 2: spread). The inflammation or tubulointerstitial nephritis (ITN) was observed with a severity score ranging from 0 to 3 (0: absent, 1: focal (> 5 % of the cortex), 2: moderate, 3: considerable). The tubular atrophy (TA) and the interstitial fibrosis (IF) were observed with a severity score ranging from 0 to 1 (0: absent, 1: present). The glomerular sclerosis (GS) was observed with a severity score ranging from 0 to 2 (0: lesion < 1 %, 1: lesion between 1 and 10 %, 2: lesion > 10 %).

S6.2. Results and discussion

An *in vivo* tolerance assay was conducted in healthy mice prior to perform additional *in vivo* efficacy studies to assess the potential toxicity of RIF associated with the dry powder for inhalation VAN/THL/25% HCO. During the tolerance study, various oral RIF concentrations (5, 7.5, 10, or 15 mg/kg) were tested in combination with endotracheal VAN/THL/25% HCO at 500 mg/kg VAN and 50 m/kg THL. None of the mice reached the humane end points defined by the ethical protocol scoring. Respiratory functions remained normal, and no abnormal behavior was observed.

The body weight of all groups (Figure S5) fluctuated during the treatment period but remained above the endpoints (i.e., no weight loss superior to 20 %, meaning for a mouse of 20g did not fall below 18 g). Initially, body weight decreased during the first week of treatment. This could be explained to the toxicity of the treatment (due to the combination of three molecules) and/or the stress generated by the cumulative administration techniques (i.e., gavage and endotracheal administration with moderate duration of general anaesthesia), leading to limiting food and water intake. It was decided to supplement mice with Solid Drink® during the second week of treatment and the recovery week. The body weight of all groups increased until the day of euthanasia (Figure S5). This demonstrated that body weight losses were reversible with an optimized nutrition, ruling out the side effects of the three drugs on body weight.

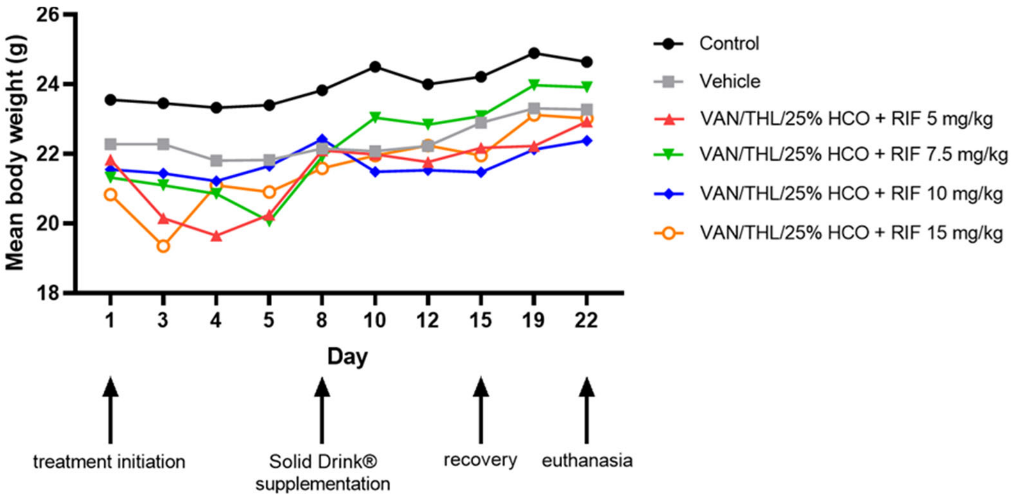

Figure S5. Mean body weight of the mice (in grams). Results are expressed as means (n = 4-6).

No macroscopic lesions were observed during the autopsy of the mice and biochemical analysis revealed no abnormalities. Only one isolated male mouse receiving VAN/THL/25% HCO with oral RIF at 15 mg/kg demonstrated a slightly higher than normal urea level. The kidneys of this animal were examined by histopathological analysis to confirm any damage. Twelve lungs' slides were stained (HE), and tissue damages were analysed and scored (Figure S6). Pulmonary congestion (Cong) was observed in all groups, with the highest severity score (score of 5). As this phenomenon also occurred in the control (group 1) and the vehicle control (group 2), these marks of stagnant blood in the tissue could be due to the euthanasia or the sacrifice technique that was made by cervical dislocation. Bronchial epithelial vacuolation (BEV) was observed only in males' mice of groups 1, 2, 3, and 4. This could represent a reversible degenerative change in the tissue. To record a potential lesion of the tissue, associated inflammation should be diagnosed, which is not the case. IAH was also observed for all groups, with a severity score

of 1. This could be traces of blood remaining in the lungs after washing, not completely removed before paraffine embedding. None of the groups displayed ALM, IAF, PH, or AB.

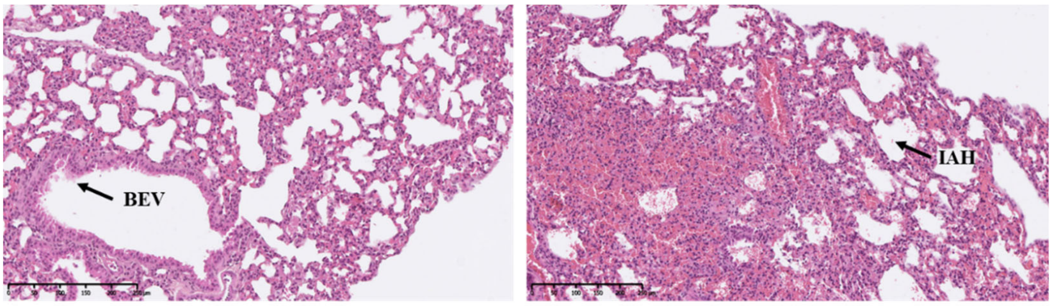

	Group 1		Group 2		Group 3		Group 4		Group 5		Group 6		Score
	F	M	F	M	F	M	F	M	F	M	F	M	0
BEV	0	3	0	1	0	1	0	1	0	0	0	0	1
Cong	5	5	5	5	5	5	5	5	5	5	5	5	2
ALM	0	0	0	0	0	0	0	0	0	0	0	0	3
IAF	0	0	0	0	0	0	0	0	0	0	0	0	4
IAH	1	0	1	0	1	0	0	1	0	1	0	1	5
PH	0	0	0	0	0	0	0	0	0	0	0	0	
AB	0	0	0	0	0	0	0	0	0	0	0	0	

Figure S6. Histopathology of lung tissue exposed to different treatments. Representative images of BEV and IAH phenomenon (HE coloration). Heat map of severity score (from 0-5) Group 1: control, Group 2: vehicle, Group 3: VAN/THL + RIF 5 mg/kg, Group 4: VAN/THL + RIF 7.5 mg/kg, Group 5: VAN/THL + RIF 10 mg/kg, Group 6: VAN/THL + RIF 15 mg/kg. "F" stands for female and "M" stands for male.

Twelve livers slides were analysed by HE and PAS staining (Figure S7). None of the groups exhibited fibrosis, fulminant hepatitis, steatosis, cholestasis, significant hepatocyte necrosis, vascular lesions (*i.e.*, thrombus and infract), or focal lesions. The control group (group 1) showed the most observations, albeit with a low severity score. Minor infiltrations in the lobular and periportal areas were noted, potentially indicating mild inflammation. Additionally, hepatocellular necrosis, the most common liver injury, was present in this group, along with abnormal fat deposition within hepatocytes (Stea) (severity score of 1). IGI were frequently observed in the group control. The vehicle group (group 2), the group treated with VAN/THL/25% HCO + 5 mg/kg oral RIF (group 3) and the group treated with VAN/THL/25% HCO + 7.5 mg/kg RIF (group 4) presented some inflammation in the periportal area (severity score of 1) and some hepatocellular necrosis (severity score of 1-2). Group 4 presented also mild IGI. The groups treated with VAN/THL + 10 mg/kg RIF (group 5) and VAN/THL + 15 mg/kg RIF (group 6) presented mild infiltrations in the tubules and the periportal with a severity score of 1-2. Depletion of glycogen (GD) was observed in groups 4, 5, and 6.

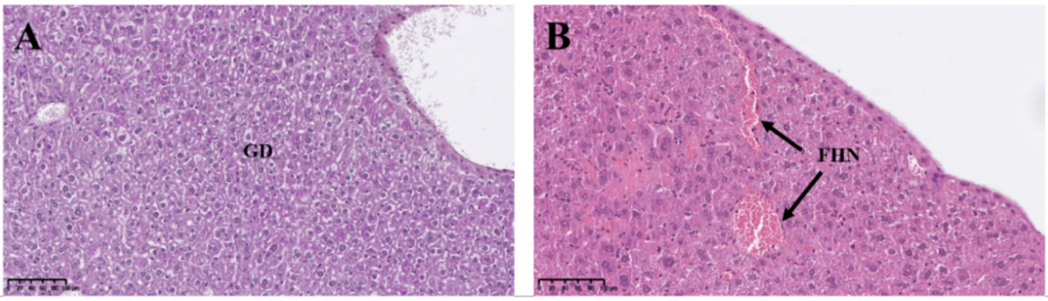

	Group 1		Group 2		Group 3		Group 4		Group 5		Group 6		Score
	F	M	F	M	F	M	F	M	F	M	F	M	0
LI	1	0	0	0	0	0	0	0	1	1	0	0-1	1
PI	1	1	0-1	1	1	1	1	0	0	1	2	0-1	2
FHN	1	0	1	0	2	1	0	1	0	0	0	0	3
MM	0	0	0	0	0	0	0	0	0	0	0	0	
Stea	0	1	0	0	0	0	0	0	0	0	0	0	
IGI	1	1	0	0	0	0	0	1	0	0	0	0	

Figure S7. Histopathology of liver tissue exposed to different treatments. Representative images of GD (with PAS coloration) (A) and FHN (with HE coloration) (B) phenomenon. Heat map of severity score (from 0-3) depending on liver tissue histopathology. Group 1: control, Group 2: vehicle, Group 3: VAN/THL + RIF 5 mg/kg, Group 4: VAN/THL + RIF 7.5 mg/kg, Group 5: VAN/THL + RIF 10 mg/kg, Group 6: VAN/THL + RIF 15 mg/kg. "F" stands for female and "M" stands for male.

Twelve kidneys' slides were stained using HE. None of the groups exhibited glomerular lesions (normal glomeruli), nor segmental or complete sclerosis. The proximal and distal convoluted tubules were contiguous, and the brush border of the proximal convoluted tubules was preserved. No tubular necrosis, tubular atrophy, or interstitial fibrosis was observed. In 3 out 12 kidneys, a few inflammatory clusters were observed focally in the interstitium of the cortex. The arterioles were normal with no arteriosclerotic lesions, and the renal arteries demonstrated no atheromatous lesions.

Conclusion

In the aim to secure the experiment that will be made on infected mice and not healthy mice (as done during the tolerance evaluation), it was chosen to continue with RIF at 7.5 mg/kg rather than 10 mg/kg as the recovery, the side effects on lung, liver and kidney were better after one week of treatment with the Solid Dink® supplementation.
